# Prognostic impact of lymphadenectomy in patients with advanced ovarian clear cell carcinoma: an ancillary analysis of the JGOG3017-A4 study

**DOI:** 10.1007/s10147-025-02926-8

**Published:** 2025-11-27

**Authors:** Satoe Fujiwara, Muneaki Shimada, Yuri Ito, Yuji Takei, Takafumi Kuroda, Koji Nishino, Tatsuyuki Chiyoda, Yasuhisa Terao, Toru Sugiyama, Aikou Okamoto

**Affiliations:** 1 Department of Obstetrics and Gynecology, Osaka Medical and Pharmaceutical University, 2-7 Daigakumachi, Takatsuki, Osaka 569-8686 Japan; 2 Department of Obstetrics and Gynecology, Tohoku University School of Medicine, 1-1, Seiryo-Machi, Aoba-Ku, Sendai, Miyagi 980-8574 Japan; 3 Tohoku University Advanced Research Center for Innovations in Next-Generation Medicine, 2-1, Seiryo-Machi, Aoba-Ku, Sendai, Miyagi 980-8573 Japan; 4 Department of Medical Statistics Research and Development Center, Osaka Medical and Pharmaceutical University, 2-7 Daigakumachi, Takatsuki, Osaka 569-8686 Japan; 5 Department of Obstetrics and Gynecology, Jichi Medical University, 3311-1 Yakushiji, Shimotsuke, Tochigi 329-0498 Japan; 6 Department of Obstetrics and Gynecology, The Jikei University School of Medicine, 3-25-8 Nishi-Shimbashi, Minato-Ku, Tokyo 105-8461 Japan; 7 Department of Obstetrics and Gynecology, Niigata University Graduate School of Medical and Dental Sciences, 757 Ichibancho, Asahimachi-Dori, Chuo, Niigata 951-8510 Japan; 8 Department of Gynecology, Niigata Cancer Center Hospital, 2-15-3 Kawaguchicho, Chuo-Ku, Niigata City, Niigata 951-8566 Japan; 9 Department of Obstetrics and Gynecology, Keio University School of Medicine, 35 Shinanomachi, Shinjuku-Ku, Tokyo 160-8582 Japan; 10 Department of Gynecology, Faculty of Medicine, Juntendo University, 2-1-1 Hongou, Bunkyo-Ku, Tokyo 113-8421 Japan; 11 Department of Obstetrics and Gynecology, St. Mary’s Hospital, 422 Tsubukuhonmachi, Kurume, Fukuoka 830-8543 Japan

**Keywords:** Ovarian cancer, Clear cell carcinoma, Systematic retroperitoneal lymphadenectomy, Prognosis

## Abstract

**Background:**

Systematic pelvic and aortic lymphadenectomy in stage IIB–IVB patients with epithelial ovarian cancer, undergoing complete abdominal macroscopic resection with normal lymph nodes, was revealed to have no prognostic significance for survival in the LION trial. However, the proportion of patients with ovarian clear cell carcinoma (OCCC) in the LION trial was only 2.2%, so the significance of systematic retroperitoneal lymphadenectomy in patients with OCCC remains unclear.

**Methods:**

We conducted an ancillary analysis of 619 patients enrolled in a randomized phase III trial (JGOG 3017) in patients with OCCC. Of these, 89 were stage IIB to IVB, underwent a complete macroscopic resection, and had no grossly enlarged lymph nodes intraoperatively. Patients were divided into two groups: group A with lymphadenectomy and group B without lymphadenectomy. The Kaplan–Meier method was used to calculate progression-free survival (PFS) and overall survival (OS) and the log-rank test and Cox proportional hazard model were used to compare the two groups.

**Results:**

Among the 89 patients, 77 (86.5%) underwent a lymphadenectomy (group A), while 12 (13.5%) did not (group B). Three-year PFS were 62.3% in group A and 58.3% in group B (*p* = 0.7705). Three-year OS were 73.0% in group A and 65.6% in group B (*p* = 0.6346). No significant differences were observed between two groups.

**Conclusion:**

This study did not demonstrate a definitive survival benefit from systematic lymphadenectomy in advanced OCCC patients with complete resection and clinically negative nodes. Given the small sample size, these results should be interpreted with caution and regarded as exploratory.

## Introduction

Epithelial ovarian cancer (EOC) is the most common cause of death from gynecological malignancies [1]. In the fifth edition of the 2020 World Health Organization classification, EOC can be grouped into five subtypes based on different histopathological features and molecular pathogeneses: high-grade serous, low-grade serous, endometrioid, mucinous, and clear cell carcinoma [2]. Particularly, mucinous or clear-cell histologies were poorly sensitive to chemotherapy and were associated with worse prognosis compared to the most frequent subtype—high-grade serous carcinoma (HGSC) [3]. In Western countries, approximately 70% of epithelial ovarian cancers are HGSC, whereas clear cell carcinoma (OCCC) is less common at 5–10% [4, 5]. Due to its rarity, OCCC has no established treatment strategy. Therefore, it is treated with the same treatment strategies as the frequent subtype, HGSC, but its biology is quite different. It is possible to speculate that different treatment strategies are needed, compared to the other different ovarian cancer histology types. The incidence of OCCC is higher in Asian women than in Western countries, at 10% in Korea and 25% in Japan [6–8]. Thus, we believe the treatment strategies for OCCC can be validated in Asian countries.

The mainstay of treatment for advanced ovarian cancer is primary surgery with the aim of a macroscopically complete resection of all visible tumors followed by chemotherapy. Surgical outcomes in ovarian cancer are classified according to the size of the largest residual tumor present after surgery, which is one of the most important prognostic factors [9]. Harter et al., in the randomized phase III LION trial, demonstrated that systematic lymphadenectomy did not improve overall survival in patients with advanced ovarian cancer who had undergone complete macroscopic resection and had no clinically suspicious lymph nodes before or during surgery [10]. However, the population of patients with OCCC in the LION trial was only 2.2%. Therefore, the significance of systemic lymphadenectomy in patients with OCCC remains unclear.

The Japanese Gynecologic Oncology Group (JGOG) 3017/Gynecological Cancer Inter Group (GCIG) trial was an international, multicenter, randomized phase III trial conducted to evaluate the efficacy of two combination regimens: the irinotecan and cisplatin regimen (CPT-P) and the paclitaxel and carboplatin regimen (TC) in patients with OCCC [11]. Over a median follow-up period of 44.3 months, no significant difference in efficacy between the two treatment arms was observed. The JGOG3017/GCIG trial enrolled 619 patients, and 171 were diagnosed with disease progression or had died. The JGOG3017/GCIG trial was the first histologic subtype-specific study that was conducted based on the International Central Pathology Review System. The data from a prospective randomized phase III trial with a large population of OCCC, a rare and refractory cancer, is significant as an extremely valuable research base.

We conducted this ancillary analysis using data from patients enrolled in the JGOG3017/GCIG trial to investigate the benefit of the addition of a systematic lymphadenectomy in patients with advanced OCCC, with clinically negative lymph nodes, who underwent complete abdominal macroscopic resection.

## Patients and methods

This ancillary study used the data from 619 patients enrolled in a randomized phase III trial of patients with OCCC, or the JGOG3017 trial. Of these, 89 patients were included in the present analysis based on case report form data, meeting the following criteria: FIGO stage IIB–IVB disease, complete macroscopic resection, and no grossly enlarged lymph nodes intraoperatively. Lymph node status was assessed based on intraoperative gross findings, not on standardized imaging or pathological confirmation across institutions. Pathological evaluation of resected lymph nodes was performed independently at each participating institution, without central review, and standardized pathological assessment protocols were not mandated across the trial. Approval for this study was granted by the ethics committee of Osaka Medical and Pharmaceutical University (approval number, No. 2022–037). The JGOG approved the utilization of clinical information from the JGOG3017 trial, which was conducted with the approval from Osaka Medical and Pharmaceutical University’s institutional review board. The requirement of informed consent was waived by the ethics committee owing to the retrospective nature of the study and the use of anonymized data.

The endpoints were the progression-free survival (PFS) and overall survival (OS) of patients with or without systematic regional lymphadenectomy. PFS was defined as the time interval from the date of the primary debulking surgery to the date of recurrence or censoring on the date of the last follow-up, whereas OS was defined at the time interval from the date of the primary debulking surgery to the date of death. Relapse was diagnosed by imaging, not by tumor markers alone.

The Kaplan–Meier method was used to estimate the survival probabilities of patients until death from any cause and recurrence. Kaplan–Meier plots were generated while stratifying by with or without systematic regional lymphadenectomy, and log-rank tests were used to assess the difference in survival between the two groups. Continuous variables are summarized as the median and the range between minimum value and maximum value. The Mann–Whitney *U* test was used to compare continuous variables and Fisher’s exact test was used to compare frequencies of categorical variables. Kaplan–Meier method was applied to calculate overall and progression-free survival. The Cox proportional hazard model was used to estimate hazard ratio (HR) and 95% confidence interval (CI). Stata version 18.0 (StataCorp, College station, TX) was used for the statistical analysis.

## Results

**Fig. 1:**
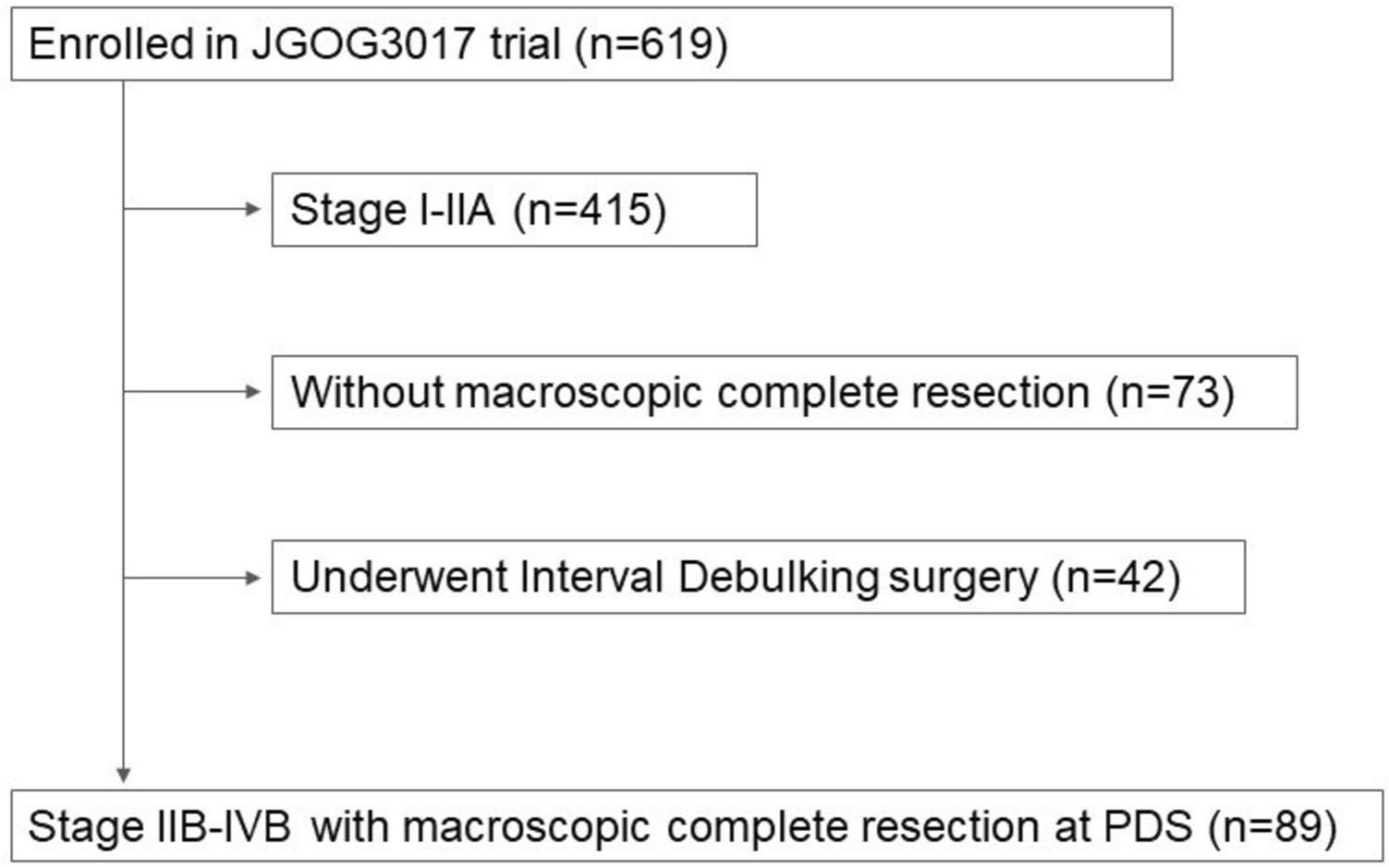
Patient selection for survival analysis

Of the 619 patients enrolled in the JGOG3017 trial, 89 were finally included in the analysis (Fig. 1). Patients were divided into two groups: Group A, who underwent systematic regional lymphadenectomy, and Group B, who did not. In total, 77 (86.5%) and 12 (13.5%) patients underwent systematic regional lymphadenectomy (Group A) or had no lymphadenectomy (Group B), respectively. The background characteristics of the patients are shown in Table 1. The median follow-up was 36.0 months (7.8–69.9 months), and the median follow-up duration of patients with lymphadenectomy was similar to those with no lymphadenectomy (36.3 months and 34.0 months, p = 0.501). As well, the patient characteristics were similar except for age. Lymph node metastasis was found in six (7.8%) of the 77 patients who underwent systematic regional lymphadenectomy by pathological examination. Of these six cases with lymph node metastasis, three (3.9%) had only pelvic lymph node metastasis, one (1.3%) had only para-aortic lymph node metastasis, and two (2.6%) had both.

**Table 1:** Clinical characteristics between lymphadenectomy and no lymphadenectomy groups

Characteristics	Lymphadenectomy group (*n* = 77)	No-lymphadenectomy group (*n* = 12)	*p*-value
Median follow-up time, median (range)	36.3 (7.8–69.9)	34.0 (14.7–54.7)	0.501
Age, yrs median (range)	55 [33–72]	66 [41–72]	0.045
< 65	69 (89.6%)	5 (41.7%)	< 0.001
65 +	8 (10.4%)	7 (58.3%)	
BMI, median (range)	20.3 [14.1–28.1]	22.1 [15.8–26.6]	0.206
< 22	56 (72.7%)	6 (50.0%)	0.174
22 +	21 (27.3%)	6 (50.0%)	
Stage			
II	46 (59.7%)	5 (41.7%)	0.348
III/IV	31 (40.3%)	7 (58.3%)	
Adjuvant chemotherapy			
TC	40 (52.0%)	7 (58.3%)	0.761
CPT-P	37 (48.0%)	5 (41.7%)	
Number of lymph nodes, median (range)	48.5 [5–89]		
Pelvic, median(range)	29 [1–68]	–	
Para-aortic, median (range)	15 [0–50]	–	
Completed adjuvant treatment			
< 6 cycles	18 (23.1%)	2 (16.7%)	1.000
6 cycles completed	59 (76.9%)	10 (83.3%)	
Ascites cytology			
Negative	29 (37.7%)	4 (33.3%)	0.401
Positive	47 (61.0%)	7 (58.3%)	
Missing	1 (1.3%)	1 (8.3%)	
Other organ resection			
No	18 (24.4%)	5 (41.7%)	0.176
Yes	59 (75.6%)	7 (58.3%)	

BMI, Body Mass Index

**Fig. 2:**
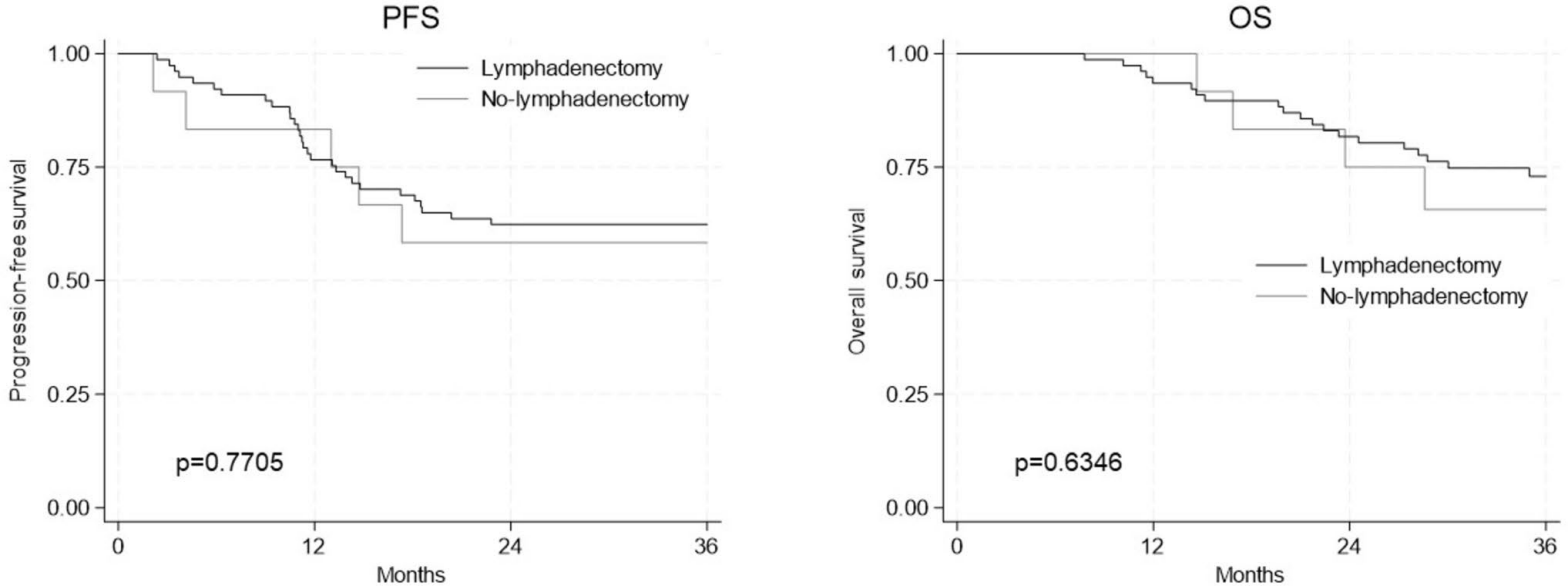
Kaplan–Meier survival curves between the lymphadenectomy group and no lymphadenectomy group. **a** Progression-free survival; **b** overall survival

The cumulative survival curves using the Kaplan–Meier method shown in Fig. 2 demonstrated the estimated 3-year PFS and 3-year OS for group A who had systematic regional lymphadenectomy and group B who did not. Three-year PFS were 62.3%for group A and 58.3% for group B (*p* = 0.7705). Three-year OS were 73.0% for group A and 65.6% for group B (*p* = 0.6346). We did not observe difference in both PFS and OS between the two groups (*p* = 0.7705 and p = 0.6346, respectively).

**Fig. 3:**
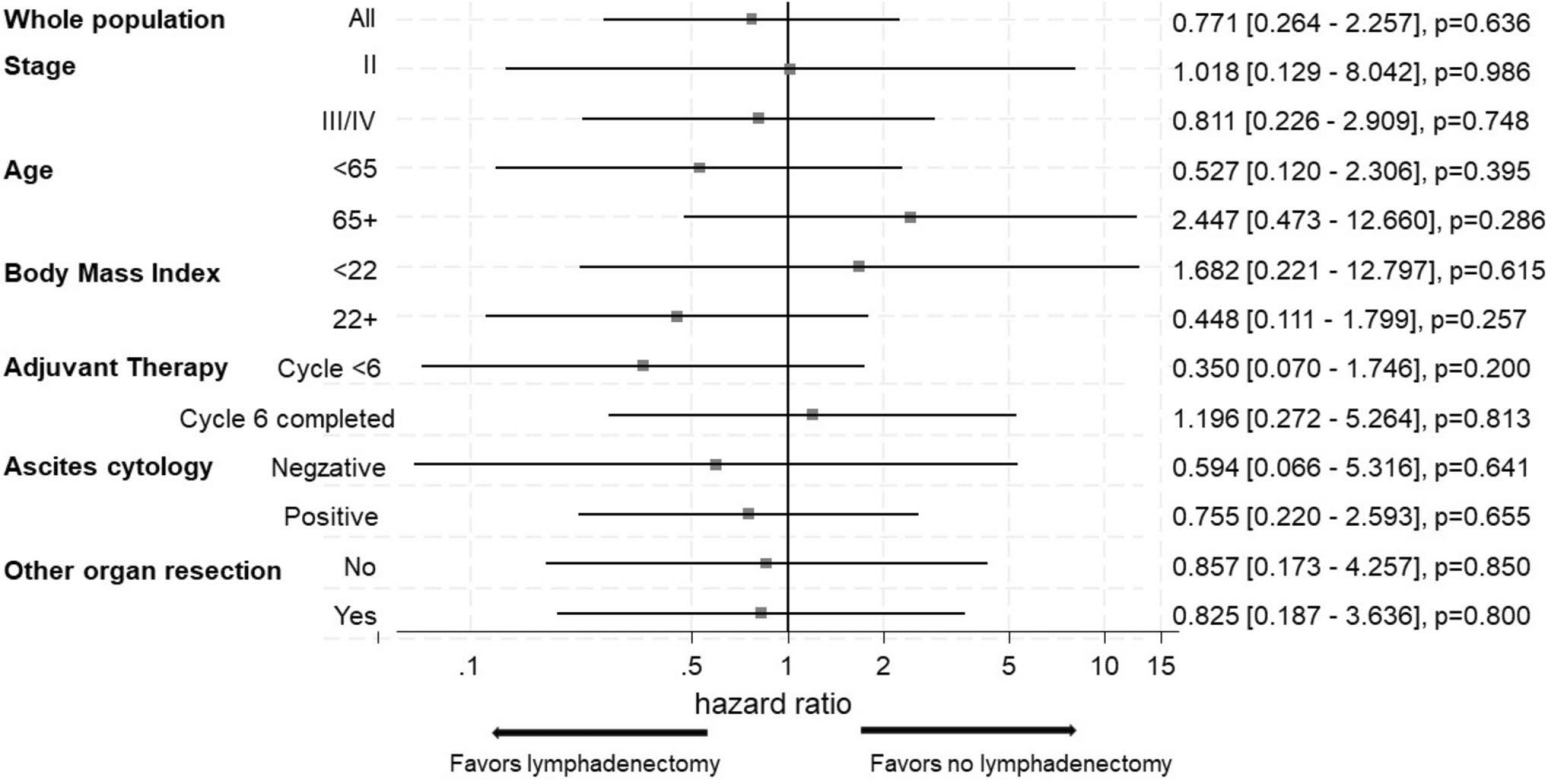
Subgroup analysis of clinicopathological factors in relation to overall survival. Hazard ratios of lymphadenectomy group to no-lymphadenectomy group estimated by the univariate Cox proportional model for OS

In Fig. 3 showing hazard ratios of lymphadenectomy group to no-lymphadenectomy group estimated by the univariate Cox proportional model for OS, there were no significant differences in all subgroups, including primary stage, ascites cytology status, and resection rate of other organs, between two groups. However, there was a favorable prognostic trend in the lymphadenectomy group for patients aged less than 65 years, with a BMI of 22 or higher, and with less than 6 cycles of adjuvant chemotherapy.

Finally, we analyzed whether there was a difference in the site of recurrence between the lymphadenectomy and no lymphadenectomy groups. The modes of tumor recurrence were classified as pelvis, upper abdominal, distant organ, or multiple site recurrence. Sites of distant metastases included lung, liver, spleen and subcutaneous tissues. As shown in Table 2a, the results for sites of recurrence were as follows: recurrence occurred in 31 of 77 patients (40.3%) with lymphadenectomy and in 5 of 12 patients (41.7%) without lymphadenectomy. Single-site recurrence was two (40%) in the no lymphadenectomy group and 10 (32%) in the lymphadenectomy group, with multiple-site recurrences, including peritoneal dissemination, in two (40%) and 19 (62%) cases, respectively. A more detailed analysis shown in Table 2b revealed 17 cases of lymph node recurrence in the lymphadenectomy group, 12 of which occurred in pelvic and para-aortic lymph nodes in the dissected area. In contrast, there were no cases of lymph node recurrence in the no lymphadenectomy group, and lymph node recurrence had not increased in this group.

**Table 2:** Sites of recurrence

(a) Form of recurrence		
	Number of patient, n (%)	
	Lymphadenectomy group (*n* = 77)	No-lymphadenectomy group (*n* = 12)
Single-site	10 (32)	2 (40)
Pelvic cavity	2 (6)	0
Upper abdomen	4 (13)	0
Distant organ	4 (13)	2 (40)
Multiple-sites	19 (62)	2 (40)
Unknown	2 (6)	1 (20)
Total	31 (100)	5 (100)

(b) Detailed site of recurrence		
	Number of patient, n (%)	
	Lymphadenectomy group (*n* = 31)	No-lymphadenectomy group (*n* = 5)
Peritoneal dissemination	12	2
Lymph node	17	0
Pelvic lymph node	3	0
Para-aortic lymph node	9	0
Mediastinal node	2	0
Supraclavicular lymph node	3	0
Pelvic cavity	5	0
Abdomen	1	0
Liver	5	1
Spleen	1	0
Lung	5	0
Pleural	3	1

## Discussion

Several retrospective studies have been reported on the prognostic impact of lymphadenectomy in advanced OCCC [12]. However, none have specifically addressed patients with complete macroscopic resection and no evidence of lymph node metastasis preoperatively or intraoperatively. Compared to HGSC, OCCC exhibits distinct biological characteristics, including a lower rate of lymphatic spread and greater resistance to chemotherapy [12]. These differences suggest that the rationale for systematic lymphadenectomy may not directly apply to OCCC. In the LION trial, which demonstrated no survival benefit from systematic lymphadenectomy in advanced ovarian cancer, only 2.2% of patients had clear cell histology [10]. Therefore, evidence specifically addressing the role of lymphadenectomy in OCCC may remain essential. Given the 7.8% lymph node involvement despite clinically negative nodes and the chemoresistance of OCCC, it remains unclear whether omitting lymphadenectomy compromises outcomes. Optimal surgical strategies for OCCC remain inadequately defined. This highlights the need to tailor treatment to the unique biology of OCCC. As described in the 2020 Japanese Society of Gynecologic Oncology guidelines for ovarian cancer, a prospective study would be required to establish the role of lymphadenectomy in advanced OCCC. [13]. However, no retrospective studies have focused specifically on patients with advanced OCCC and clinically negative lymph nodes to provide a foundation for such prospective research. To our knowledge, this is the first study to evaluate the prognostic impact of systematic lymphadenectomy exclusively in patients with advanced OCCC who underwent complete macroscopic resection and had no intraoperative evidence of lymph node metastasis—a cohort analogous to that studied in the LION trial.

Although the results of an earlier study showed that complete surgical staging involving systematic lymphadenectomy appeared to improve survival in patients with stage I OCCC [14], additional recent research has shown no such benefit [15]. Furthermore, in advanced OCCC, especially in cases with a macroscopically complete resection and with clinically negative lymph nodes, the prognostic impact of lymphadenectomy is unclear. Liu et al. reported, in a retrospective study using SEER and Chinese registry data, that the performance of lymphadenectomy in patients with OCCC did not demonstrate a significant impact on survival for either early or advanced stage patients [16]. The results of these studies are not limited to patients with macroscopically complete resection, as they also include patients with macroscopically residual tumors and those with clinically positive nodes. Gao et al. also reported that lymphadenectomy does not significantly impact survival in OCCC [17]. Furthermore, a sub-analysis of this study showed similar results in patients with clinically negative lymph nodes, but the analysis was performed for both early and advanced stage cases. In the surgical approach of OCCC, the significance to analyze lymphadenectomy in the early stage and advanced stage is different.

For OCCC patients with early stage, the frequency of lymph node metastasis is much lower than with other tumor subtypes, according to previous studies. Several previous studies have reported low rates of lymph node metastasis in patients with OCCC and at an early stage, from 0 to 11% [17–21]. Mahdi et al., in 2013 identified nearly 1900 OCCC patients restricted to the ovary through the SEER program. Among the 1359 cases that underwent lymphadenectomy, only 61 (4.5%) had positive lymph nodes [21]. Heitz et al. also showed that 3.6% of OCCC patients with clinically negative lymph nodes had lymph node metastasis, while the rate was 71.6% in patients with high-grade serous ovarian cancer [18]. On the other hand, Gao et al. reported high rates of lymph node metastasis of 58.8% in pT3 cases, but they included cases with clinically positive lymph nodes in these pT3 cases [17]. There are no reports on the rate of lymph node metastasis in advanced OCCC with clinically negative lymph nodes. In our study, the rate of lymph node metastasis in advanced OCCC with clinically negative lymph nodes was 7.8%, a very low rate compared to the 57% in the LION study, in which 85% of cases were HGSC [10]. Matsuo et al. reported that adequate dissection is achieved with at least 8–12 dissected lymph nodes in ovarian cancer [22]. The median number of lymph nodes removed in this study was 48.5 (range, 5–89), which we believe ensures the quality of skill of lymphadenectomy; the low rate of lymph node metastasis even in advanced OCCC with clinically negative lymph nodes may be the reason why lymphadenectomy does not have an impact on prognosis.

Interestingly, our study showed no difference in the frequency of single or multiple-site recurrences between the lymphadenectomy and no-lymphadenectomy groups. Peritoneal dissemination was the most common pattern of recurrence in both groups (38.7% vs. 40%), consistent with previous reports [23]. Notably, lymph node recurrence occurred only in the lymphadenectomy group (48%), and all were within the dissected regions. While the median number of dissected lymph nodes was high (48.5), suggesting that the extent and quality of lymphadenectomy were likely maintained, we acknowledge that there was no direct method to validate the adequacy or completeness of the procedure across institutions. This represents an important limitation of our study. Nevertheless, the occurrence of nodal recurrences within the dissected areas may imply that, in OCCC, even technically appropriate lymphadenectomy might be insufficient to fully eliminate microscopic lymphatic disease. This observation highlights the need for further investigation into the biological behavior of OCCC and the limitations of current surgical strategies.

Although this analysis was based on data from a randomized phase III trial, several limitations must be acknowledged. Additional limitations of this study include the lack of criteria for preoperative and intraoperative lymph node metastasis assessment criteria, the differences in the choice of adjuvant chemotherapy regimens at the time of this phase III trial, and the potential differences in the quality of care between the two treatment groups due to lack of randomization, e.g., quality of surgical skill, or surgical material and peri-operative care, and potential differences in patient’s background between two treatment groups, e.g., undocumented factors that can lead physicians to avoid lymphadenectomy. These limitations warrant caution in interpreting the results. To our knowledge, this study is one of the first to explore the prognostic impact of systematic lymphadenectomy in advanced OCCC patients with complete macroscopic resection and no clinical evidence of lymph node metastasis. To better understand surgical decision-making and clarify the role of lymphadenectomy in this population, further retrospective analyses with detailed clinical background data are warranted, which may ultimately inform future prospective trials. Moreover, the observed age imbalance between groups (median age 55 vs. 66) suggests possible selection bias, likely reflecting clinical decisions on surgical eligibility. Due to the small sample size and lack of detailed background data (e.g., performance status, comorbidities), we could not perform multivariable or propensity score-adjusted analyses. These limitations should be carefully considered. A separate retrospective study is underway to clarify surgical decision-making factors. Additionally, the extent of lymphadenectomy (e.g., pelvic and/or para-aortic) was not standardized within the JGOG3017 protocol and may have varied between institutions. This variability could influence both staging accuracy and survival outcomes, and represents another limitation of this retrospective analysis. Although the JGOG3017 trial was primarily conducted in Japan and the majority of patients were Japanese (93.5%), a small proportion (6.5%) were non-Japanese. Therefore, while our analysis population predominantly reflects Japanese clinical practice, the findings may have broader relevance, though generalizability should still be approached with caution. However, given that the biological characteristics of OCCC—such as low lymphatic dissemination and chemoresistance—are shared across ethnic groups, the surgical implications may be applicable to OCCC cases regardless of regional prevalence.

Importantly, our study focused on a subset of patients analogous to those included in the LION trial—those with FIGO stage IIB–IVB disease, no gross lymphadenopathy, and complete macroscopic resection. This was a deliberate design choice, as the applicability of the LION trial findings to OCCC remains uncertain given the limited representation of this histologic subtype. Therefore, our results should be interpreted in the context of this specific population, rather than generalized to all advanced OCCC patients. In addition, the limited sample size in this analysis may in part be attributed to the inherent clinical characteristics of OCCC. It is well established that OCCC is typically diagnosed at an early stage, and the proportion of patients with advanced-stage disease (FIGO stage III or higher) is relatively low, as previously reported [12]. This trend was also observed in the JGOG3017 phase III trial [11], despite its large-scale, multicenter design. Furthermore, when restricting the analysis to patients analogous to the LION trial cohort—those with advanced-stage disease, no gross lymphadenopathy, and complete macroscopic resection—the number of eligible patients became even more limited. Although our analysis used data from a randomized phase III trial, it should be emphasized that this ancillary study was retrospective in nature and not powered to detect small-to-moderate differences in survival outcomes. In particular, the number of patients in the non-lymphadenectomy group was only 12, which severely limits the statistical power and raises the possibility of a type II error. Therefore, the lack of statistically significant differences in survival should not be interpreted as definitive evidence of no benefit.

In conclusion, an ancillary analysis using data from JGOG3017 showed no statistically significant survival benefit from regional lymphadenectomy after complete resection in patients with advanced OCCC without clinically evident lymph node metastasis. However, the number of eligible cases was very small because advanced OCCC is rare. The limited cases in the non-dissection group and the retrospective design reduced statistical power, so the non-significant results may reflect a Type II error. These findings should not be interpreted as evidence against regional lymphadenectomy. Clarifying the role of lymphadenectomy in surgical management for advanced OCCC remains a critical research issue. This exploratory study represents a first step. The efficacy of node sampling for OCCC should be evaluated by a Japanese clinical trial group. Further retrospective data collection is ongoing, and a future prospective study in Japan is strongly encouraged.

## Abbreviations

OCCC: Ovarian clear cell carcinoma
EOC: Epithelial ovarian cancer
HGSC: High-grade serous carcinoma
JGOG: Japanese Gynecologic Oncology Group
GCIG: Gynecological Cancer Inter Group
HR: Hazard ratio
CI: Confidence interval
PFS: Progression-free survival
OS: Overall survival
FIGO: International Federation of Gynecology and Obstetrics
